# Collimator position optimization for proton minibeam radiation therapy

**DOI:** 10.21203/rs.3.rs-8768924/v1

**Published:** 2026-02-16

**Authors:** Nimita Shinde, Yuting Lin, Hao Gao

**Affiliations:** Department of Radiation Oncology, University of Texas Southwestern Medical Center, Dallas, TX, USA

**Keywords:** proton minibeam radiotherapy (pMBRT), multi-slit collimator (MSC), mixed integer programming (MIP)

## Abstract

Proton minibeam radiation therapy (pMBRT) employs spatially fractionated dose distributions to reduce normal tissue toxicity. A key component is the multi-slit collimator (MSC), which shapes the beam into narrow, spatially separated minibeams. Small lateral shifts of the MSC relative to the beam direction can substantially alter peak-valley dose patterns, target coverage, and organs-at-risk (OAR) sparing, making MSC positioning a critical planning parameter. We develop a novel collimator position optimization (CPO) algorithm for pMBRT that allows independent lateral shifts of the MSC at each beam angle to improve plan quality. The problem is formulated as a mixed-integer programming (MIP) model that jointly optimizes MSC positions and spot intensities. Binary variables select candidate lateral shifts per beam angle, while continuous variables represent spot intensities. The resulting non-convex problem is solved using an augmented Lagrangian framework with iterative convex relaxation and alternating direction method of multipliers (ADMM) decomposition. In three clinical cases, the proposed method achieved near-optimal solutions with substantially reduced computation time compared to exhaustive enumeration (e.g., 700 s vs. 15,000 s for an abdominal case). Allowing multiple MSC positions per beam angle led to consistent dosimetric improvements, particularly in OAR sparing; for example, mean oral cavity dose in a head-and-neck case decreased from 6.5 Gy to 4.6 Gy. MSC position optimization enhances pMBRT plan quality and can be efficiently integrated into clinical treatment planning.

## 1. Introduction

Proton minibeam radiation therapy (pMBRT) [1,2] is an emerging treatment modality that employs arrays of sub-millimeter proton beamlets, creating highly heterogeneous entrance dose distributions while achieving a near-uniform target dose at tumor depth through multiple Coulomb scattering and beam overlap. A key component of pMBRT is the use of multi-slit collimator (MSC) [2], a device that partitions the proton beam into an array of narrow, spatially separated minibeams. These beamlets, spaced a few millimeters apart, produce distinct radiobiological effects compared to conventional broad-beam delivery [2–5]. By adjusting beam shape and spatial patterning, MSC plays a central role in achieving the high spatial modulation characteristic of pMBRT.

The design and application of MSC in pMBRT have been investigated in both preclinical and computational studies [2,5–10]. Other studies have examined how MSC parameters such as slit width, center-to-center (ctc) spacing, and edge sharpness influence lateral dose profiles and spatial fractionation characteristics [11–14]. More recently, [12] introduced a joint dose and PVDR optimization framework combined with a multi-collimator strategy, in which MSC with different ctc distances are selected across different beam angles to improve both OAR sparing and target PVDR. Further, [14] proposed an MIP-based framework to optimally select MSC with varying ctc distances from a predefined candidate set at every beam angle.

Despite these advances, most existing pMBRT studies (including [12,14]) assume fixed lateral MSC positions for each beam angle, limiting the ability to exploit MSC positioning as an additional degree of freedom in inverse treatment planning. In this work, “MSC position” refers to the lateral placement of the MSC, achieved by shifting the MSC sideways (laterally) relative to the beam direction by a fraction of the ctc distance. The assumption of fixed MSC positions may be suboptimal in clinical scenarios, as MSC positioning determines the spatial location of the minibeams within the patient. Because minibeams create fractionated high-dose patterns, the relative position of the MSC dictates which anatomical regions receive peak doses along each beam path.

If the MSC is not optimally positioned, certain tumor regions may be consistently blocked across all beam angles, leading to underdosing, while other target regions may receive overlapping radiation from multiple angles, leading to over exposure and non-uniform target coverage. Additionally, for anatomically complex targets, particularly when the target lies near critical OAR from multiple directions, adjusting MSC positions can also improve OAR sparing.

This work proposes a mixed-integer programming (MIP) framework, collimator position optimization (CPO), to optimize MSC positions independently for each beam angle. Binary decision variables select MSC positions from a set of candidate lateral shifts, and the model simultaneously determines optimal spot intensities using an inverse planning approach. Candidate shifts are chosen as fractions of the ctc spacing, since shifts larger than the ctc distance are equivalent (e.g., for a 4 mm ctc distance, shifts of 1 mm and 5 mm are effectively the same). A second MIP model extends this formulation to allow multiple MSC with different positions to be used at the same beam angle, enabling multiple independent minibeams per direction. In this formulation, the total number of MSC, and thus the total number of beams in the plan, is upper bounded. The two frameworks, CPO-S and CPO-M, correspond to the use of single or multiple MSC per beam angle, respectively. The proposed models are evaluated using three clinical cases, with assessments of both dosimetric quality and computational performance. Optimality of CPO-S is further validated by exhaustive enumeration of all possible MSC configurations, confirming that the MIP framework consistently identifies nearly optimal configurations.

## 2. Methods

### 2.1. Problem definitions

Two MIP formulations are proposed for MSC position optimization problem.

**Model 1:** The first formulation selects a single optimal MSC position from a set of K candidate positions for each of the B beam angles. Binary variables $y_{kj}$ indicate the active MSC position k for each beam angle j, and the spot intensities are computed as:

(1) $$\underset{x,y}{min}\;f(d)\;\text{s.t.}\;\text{d}=\sum_{j=1}^{B}\left(\sum_{k=1}^{K}A_{kj}y_{kj}\right)x_{j},\;x_{j}\in \{0\}\cup [G,+\infty \}\;\forall j\in [B]\;\sum_{k=1}^{K}y_{kj}=1\;\forall j\in [B]\;y_{kj}\in \{0,1\}\;\forall j\in [B],\forall k\in [K].$$

**Model 2:** The second formulation extends Model 1 by allowing multiple MSC with different positions to be used at the same beam angle. A global upper bound N is imposed on the total number of MSC used across all angles:

(2) $$\underset{x,y}{min}f(d)\;\text{s.t. d}=\sum_{j=1}^{B}\left(\sum_{k=1}^{K}A_{kj}y_{kj}\right)x_{j},\;x_{j}\in \{0\}\cup [G,+\infty \}\;\forall j\in [B]\;\sum_{k,\text{j}}y_{kj}=N\;y_{kj}\in \{0,1\}\;\forall j\in [B],\forall k\in [K].$$

In both formulations, $A_{kj}$ is the dose deposition matrix for each beam angle j and each candidate MSC position k, and G is the minimum monitor unit (MMU) value. The continuous decision variable $x_{j}$ corresponds to the spot intensity vector for each beam angle j=1,…,B to be optimized, and the binary decision variable $y_{kj}$ determines whether position k is chosen for the MSC at beam angle j.

Each candidate position represents a distinct lateral shift of the MSC relative to the beam direction, with each shift corresponding to a unique fraction of the ctc distance. A 0 mm shift corresponds to the default MSC position, while +1 mm or −1 mm shifts indicate moving the MSC laterally to the right or left, respectively, by 1 mm. Allowing MSC positions to vary independently across beam angles introduces additional degrees of freedom in the treatment planning process, providing the potential to improve target dose uniformity while minimizing OAR exposure.

In both formulations, the first constraint defines the dose distribution d based on the selected MSC positions, and the second constraint enforces the MMU requirement [15,16], ensuring the treatment plan deliverability. The third and fourth constraints together impose a binary restriction on $y_{kj}$. In Model 1, the third constraint enforces selection of exactly one MSC position per beam angle. In Model 2, the third constraint enforces exactly N MSC are chosen across all beam angles, allowing multiple MSC to be assigned to the same beam angle if beneficial. This enables multiple distinct minibeams to be generated from a single angle.

Finally, the objective in both models minimizes the least squared error between the prescribed and delivered dose as well as the error in DVH-based constraints and is given as

$$f(d)=\sum_{i=1}^{N_{1}}\frac{w_{1i}}{n_{i}}{\left\|d_{\Omega_{1i}}-b_{1i}\right\|}_{2}^{2}+\sum_{i=1}^{N_{2}}\frac{w_{2i}}{n_{i}}{\left\|d_{\Omega_{2i}}-b_{2i}\right\|}_{2}^{2}+\frac{w_{3}}{n}{\left\|d_{\Omega_{3}}-b_{3}\right\|}_{2}^{2}\;+\sum_{i=1}^{N_{4}}\frac{w_{4i}}{n_{i}}{\left\|d_{\Omega_{4i}}-b_{4i}\right\|}_{2}^{2}+\sum_{i=1}^{N_{5}}\frac{w_{5i}}{n_{i}}{\left\|d_{\Omega_{5i}}-b_{5i}\right\|}_{2}^{2}.$$

- **Target dose matching:** The first term represents $N_{1}$ least square error terms measuring the deviation between the delivered dose $d_{\Omega_{1i}}$ and the prescribed dose $b_{1i}$ for the target and OAR. Here, $\Omega_{1i}$ is the set of voxels where the delivered dose differs from the prescribed dose.
- **DVH-max constraint for OAR:** The second term in f(d) enforces $N_{2}$ dose volume histogram (DVH)-max constraints [17,18] for OAR, ensuring that at most fraction p of voxels in OAR i receive a dose more than $b_{2i}$. The set $\Omega_{2i}$ is defined to include voxels indices that violate the constraint. More formally, if $d^{'}$ denotes the dose d sorted in descending order and if $n_{i}$ is the number of voxels in OAR i, then $\Omega_{2i}=\left\{j|j\geq p\times n_{i}\right\}$ when $d_{p\times n_{i}}^{'}\geq b_{2i}$. Thus, the second term in f(d) minimizes the error between the delivered dose $d_{\Omega_{2i}}$, and the DVH-max dose $b_{2i}$ for voxel indices that violate the constraint.
- **DVH-min constraint for the target:** The third term defines a DVH-min constraint [17,18] for the target, to ensure that at least a fraction p of all target voxels receive at least $b_{3}$ amount of dose. Sorting the dose d in descending order as $d^{'}$ and letting n be the total number of voxels in the target, the active index set, $\Omega_{3}$, for this constraint can be defined as $\Omega_{3}=\{j|j\leq p\times n\}$ if $d_{p\times n}^{'}\leq b_{3}$, where $d^{'}$ is the dose d sorted in descending order, and n is the number of voxels in the target. Thus, the third term in f(d) defines DVH-min constraint for target voxels violating the constraint.
- **D-max constraint for OAR:** The fourth term in f(d) defines the penalty for any OAR voxels that violate the D-max (dose-max) constraint. For any OAR i, the D-max constraint penalizes any voxel in OAR i that receives dose more than $b_{4i}$. An active index set is defined as $\Omega_{4i}=\left\{j\in \left[n_{i}\right]|d_{j}\geq \right.\left.b_{4i}\right\}$, which is non-empty when the constraint is violated. Thus, the error term ${\left\|d_{\Omega_{4i}}-b_{4i}\right\|}_{2}^{2}$ defines the least square penalty for violating the D-max constraint.
- **D-mean constraint for OAR:** The fifth term in f(d) defines the penalty for violation of the D-mean (dose-mean) constraint. For an OAR i, this constraint defines the average dose delivered to all voxels in OAR i to be less than or equal to $b_{5i}$. If the D-mean constraint is satisfied, then the set $\Omega_{5i}$ is empty. Otherwise, $\Omega_{5i}=\left[n_{i}\right]$, i.e., the active index set consists of all voxels in OAR i.

### 2.2 CPO-S: Solution methodology for Eq. (1)

To efficiently solve the proposed model, Eq. (3) first reformulates Eq. (1) using auxiliary variable z as

(3) $$\underset{x,y}{min}\;f(d)\;\text{s.t.d}=\sum_{j=1}^{B}\left(\sum_{k=1}^{K}A_{kj}y_{kj}\right)x_{j},\;x_{j}=z_{j}\forall j\in B\;z_{j}\in \{0\}\cup [G,+\infty \}\;\forall j\in [B]\;\sum_{k=1}^{K}y_{kj}=1\;\forall j\in [B]\;y_{kj}\in \{0,1\}\;\forall j\in [B],\forall k\in [K].$$

The augmented Lagrangian of Eq. (3) is then defined as

(4) $$\underset{x,y}{min}\;f\left(\sum_{j=1}^{B}\left(\sum_{k=42}^{K}A_{kj}y_{kj}\right)x_{j}\right)+\frac{\mu_{1}}{2}\sum_{j\in [B]}{\left\|x_{j}-z_{j}+\lambda_{1j}\right\|}_{2}^{2}+\frac{\mu_{2}}{2}\sum_{j\in [B]}{\left(\sum_{k=1}^{K}y_{kj}-1+\lambda_{2j}\right)}^{2}\;\text{s. t.}\;z_{j}\in \{0\}\cup [G,+\infty \}\;\forall j\in [B],\;y_{kj}\in \{0,1\}\;\forall j\in [B],\forall k\in [K].$$

The resulting inverse optimization problem (Eq. (4)) [19–28] can now be solved using iterative convex relaxation (ICR) [29,30] and the alternating direction method of multipliers (ADMM) [31,32]. The iterative method sequentially updates each primal decision variable while keeping others fixed. The binary variable is updated by solving quadratic unconstrainted binary optimization problem using MATLAB-based quantum inspired ‘qubo’ solver. Algorithm 1 outlines the proposed solution methodology for Eq. (4).

**Algorithm 1:** CPO-S: Optimization method for solving Eq. (4)

**Input:** Choose input parameters $\mu_{1},\mu_{2}$ **Initialization:** Randomly initialize x,y. Choose number of iterations T. Set $z_{j}=x_{j},\lambda_{1j}=\lambda_{2j}=0$. For t=1,…,T **Update active index sets:** Update active index set for all DVH constraints as described in Section 2.1. **Update primal variables:** Sequentially update the primal variables $x_{j},z_{j},y_{kj}$ by fixing all other variables and solving the resulting minimization problem for each. **Update dual variables:** Perform the following updates: $$\lambda_{1j}=\lambda_{1j}+x_{j}-z_{j}$$ $$\lambda_{2j}=\lambda_{2j}+\sum_{k=1}^{K}y_{kj}-1.$$ **Output:** x,y

#### Updating primal variables in Algorithm 1 (Step 4b):

1. Updating $x_{j}$ : For each j=1,…,B, fix all variables except $x_{j}$ in Eq. (4). The resulting minimization problem is unconstrained in $x_{j}$, and thus, the optimal value of $x_{j}$ is obtained by taking first-order derivative of the objective with respect to $x_{j}$, setting it to zero, and solving the resulting linear system.
2. Updating $z_{j}$: For each j=1,…,B, fix all variables except $z_{j}$ in Eq. (4). The solution to the resulting minimization problem has a closed form solution, obtained via soft thresholding:
$$z_{j}=\left\{\begin{array}{rr} max\left(G,x_{j}-\lambda_{1j}\right), & \text{if}\;x_{j}-\lambda_{1j}\geq G/2\\ 0, & \text{otherwise.} \end{array}\right.$$
3. Updating $y_{kj}$: Fixing all variables in Eq. (4) except the binary variable $y_{kj}$ results in a quadratic unconstrained binary optimization (QUBO) problem, a well-studied problem in quantum computing. In this work, the QUBO problem is solved using ‘qubo’, a MATLAB built-in solver, to find the optimal value of $y_{kj}$’s at each iteration.

After Algorithm 1 generates near-optimal MSC positions, a final inverse optimization is performed using these positions to further refine spot intensities.

### 2.3 CPO-M: Solution methodology for Eq. (2)

The solution methodology for Eq. (2) follows the same overall approach as that for Eq. (1) described in Section 2.2. An auxiliary variable z is introduced, and Eq. (2) is reformulated. The augmented Lagrangian of the reformulated problem is given by

(5) $$\underset{x,y}{min}f\left(\sum_{j=1}^{B}\left(\sum_{k=1}^{K}A_{kj}y_{kj}\right)x_{j}\right)+\frac{\mu_{1}}{2}\sum_{j\in [B]}{\left\|x_{j}-z_{j}+\lambda_{1j}\right\|}_{2}^{2}+\frac{\mu_{2}}{2}{\left(\sum_{k,\text{j}}y_{kj}-N+\lambda_{2}\right)}^{2}\;\text{s. t.}\;z_{j}\in \left\{0\right\}\cup \left[G,+\infty \right\}\;\forall j\in \left[B\right],\;y_{kj}\in \left\{0,1\right\}\;\forall j\in \left[B\right],\forall k\in \left[K\right].$$

Eq. (5) can be solved using Algorithm 1 with the following minimal modifications:

- **Updating y:** The binary variable $y_{kj}$ is again updated by solving the QUBO problem using the MATLAB-based ‘qubo’ solver. However, in this case, the objective function differs slightly from Eq. (4) because of the change in the last term of the augmented Lagrangian Eq. (5).
- Updating $\lambda_{2}$: The dual variable $\lambda_{2}$ is updated as $\lambda_{2j}=\lambda_{2j}+\sum_{k,j}y_{kj}-N$.

### 2.4 Materials

The impact of optimally selecting MSC positions, as well as the effectiveness of the proposed method in identifying such positions, was evaluated using three clinically relevant test cases: two head-and-neck (HN) cases and one abdomen case. This work used retrospective, fully anonymized clinical datasets and did not involve active participation of human subjects. In all cases, MSC with a 4 mm center-to-center (ctc) distance and 0.4 mm slit width were used. The HN cases employed four beam angles (B=4) at 45°, 135°, 225° and 315°, while the abdomen case employed three beam angles (B=3) at 0°, 120° and 240°.

For treatment plan optimization, four available candidate MSC positions (K=4), corresponding to lateral shifts of 0 mm, 1 mm, 2 mm, and 3 mm relative to the default MSC position (0 mm shift), were considered at all angles. In Eq. (2), N was set to four and three for HN and abdomen cases, allowing a total of four and three MSC, respectively to be used in the plan, with the possibility of assigning multiple MSC positions to the same angle, thereby generating multiple independent beams from that angle.

To evaluate the optimality of the optimization method (CPO-S) that solves Eq. (1), a reduced set of three candidate MSC positions was considered at each beam angle, corresponding to shifts of 0 mm, 1 mm, and 2 mm. This yielded 81 possible MSC configurations in the HN cases, and 27 possible configurations in the abdomen case. For each configuration, a complete inverse treatment planning optimization was performed, and exhaustive enumeration of all configurations was used to identify the globally optimal solution. These results enabled direct comparison with solutions obtained using the proposed optimization framework.

Within the CPO-S framework, the binary subproblem was solved using two different approaches: (i) the built-in MATLAB ‘qubo’ solver (denoted CPO-S-Q), corresponding to the method described in Section 2.2, and (ii) a classical MIP solver (denoted CPO-S-Cl). These two variants were evaluated alongside exhaustive enumeration to assess solution optimality and computational performance. The corresponding results are presented in Section 3.1.

Five planning approaches were evaluated in this study: (1) intensity modulated proton therapy without MSC (IMPT), (2) conventional pMBRT with fixed (0 mm shift) MSC positioning (Conv), (3) joint dose and PVDR optimization (JDPO) as proposed in [12], (4) single-MSC optimization using MIP (CPO-S), and (5) multiple-MSC optimization using MIP (CPO-M). All planning approaches were optimized using the same dose-based objective function, f(d), and identical weighting parameters. Differences in plan quality therefore arise solely from the differences in MSC configuration: fixed MSC positioning in the conventional approach, optimized MSC positioning in CPO-S and CPO-M, MSC selection combined with explicit PVDR optimization in JDPO, and the absence of MSC in IMPT. This design ensures a controlled and fair comparison across all methods.

Dose deposition matrices for each beam angle and each candidate MSC position were generated using matRad [33], with a spot width of 0.4 mm and a dose calculation grid resolution of 1×1×3 mm^3^. CTV-based planning was performed using clinically defined constraints for all test cases. All treatment plans were normalized such that at least 95% of the target volume received 100% of the prescribed dose. Plan quality was evaluated using the following metrics: (a) maximum dose delivered to the tumor $\left({\text{D}}_{\text{max}}\right)$, (b) mean and maximum doses delivered to OAR, (c) conformity index (CI), and (d) PVDR evaluated on beam’s-eyeview (BEV) slices for each angle. The CI was calculated as ${\left({\text{V}}_{100}\right)}^{2}/\left({\text{V}}_{\text{T}}*\text{V}\right)$, where ${\text{V}}_{100}$ is target volume receiving at least 100% of the prescribed dose, ${\text{V}}_{\text{T}}$ is the total target volume, and V is the volume that receives at least 100% of the prescribed dose. The PVDR at each slice was calculated as D_20_/D_80_, where D_20_ and D_80_ are the doses delivered to at least 20% and 80% of the volume under consideration. The normalized maximum dose D_max_ is calculated as (D/D_p_)×100%, where D is the maximum dose delivered to the tumor and D_p_ is the prescribed dose.

## 3. Results

### 3.1 Optimality of MIP method: CPO-S

The optimality of the proposed MIP-based method (CPO-S) was evaluated by comparing two variants: (i) the heuristic approach using MATLAB’s built-in qubo solver (CPO-S-Q, as described in Section 2.2), and (ii) a classical MIP solver (CPO-S-Cl), against exhaustive enumeration of all possible MSC configurations for Eq. (1). For each case, three candidate shifts per angle were considered, resulting in 81 possible configurations for the HN cases and 27 configurations for the abdomen case. For each configuration, the inverse optimization problem was solved, and the configurations were ranked in ascending order of the objective function value, with rank 1 representing the best (lowest) value.

**Figure 2:**
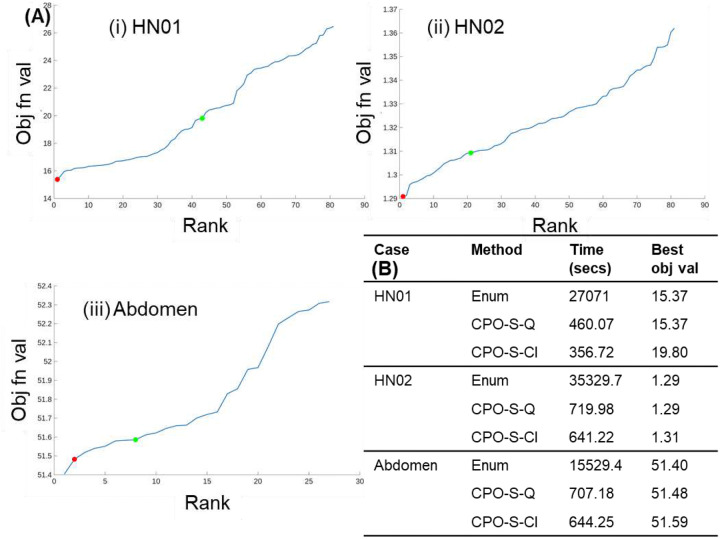
(A) Objective function values for all combinations of three MSC shifts across beam angles, ranked in ascending order. Red and green markers indicate the CPO-S-Q and CPO-S-Cl solutions, respectively. CPO-S-Q ranks first for HN01 and HN02, and second for the abdomen case. (B) Computation time and best objective function values for exhaustive enumeration versus the two CPO-S variants (Algorithm 1).

Figure 2(A) shows the distribution of objective values, with the solutions from CPO-S-Q and CPO-S-Cl marked for each case with red and green dots respectively. In the HN01 and HN02 cases, CPO-S-Q identified the globally optimal configuration (rank 1 of 81), with runtimes of approximately 460 and 720 seconds, respectively, much faster than exhaustive enumeration (27,000 and 35,000 seconds). Additionally, although CPO-S-Cl generated the solutions more quickly than CPO-S-Q (356 and 640 seconds for HN01 and HN02 cases), it failed to identify near-optimal configurations, indicating reduced robustness. This is because the overall optimization problem is non-convex, and so, the heuristic CPO-S-Q method appears more effective at escaping poor local minima than the classical MIP solver, which often becomes trapped.

For the abdomen case, the CPO-S-Q solution ranked second overall, with objective values within 1% of the global optimum and runtime of approximately 700 seconds, respectively, compared to more than 15,000 seconds for the enumerative approach. In contrast, CPO-S-Cl did not yield competitive solutions for the abdomen case as seen in Figure 2(B). Furthermore, CPO-S-Q, despite being heuristic, produced consistent results when initialized with the same parameters $\left(\mu_{1},\mu_{2}\right)$ and decision variables (x,y), suggesting stable convergence behavior.

These results confirm that the CPO-S method using the built-in MATLAB ‘qubo’ solver can achieve optimal or near-optimal solutions with significantly reduced computation times compared to enumeration, making it a practical and computationally efficient approach for determining optimal MSC configurations and clinical treatment planning.

### 3.2 Comparison of treatment plan quality

#### 3.2.1 Comparison with IMPT

Across all evaluated cases, the proposed methods (CPO-S and CPO-M) demonstrate distinct trade-offs relative to IMPT planning. As expected, IMPT consistently achieves lower maximum OAR dose compared to SFRT-based approaches due to homogenous nature of IMPT. In contrast, spatially fractionated techniques produce localized dose peaks by design. However, despite these higher peak (maximum) doses in normal tissues compared to IMPT, CPO-M yields lower mean doses to most of the OAR than IMPT in all cases, indicating improved overall normal tissue sparing. For example, in HN01, the mean dose to oral cavity decreases from 5.76 Gy (IMPT) to 4.64 Gy (CPO-M) method. This reduction in mean OAR dose is particularly pronounced in anatomically complex cases, such as HN, where spatial modulation can be more effectively exploited.

In addition, SFRT-based plans achieve substantially higher PVDR values than IMPT across all cases, confirming the presence of a spatially fractionated dose regime that is absent in IMPT. Both CPO-S and CPO-M provide equivalent PVDR values for all cases. However, between the two proposed approaches, CPO-M consistently provides the lowest OAR mean doses among all SFRT methods, reflecting the added flexibility afforded by allowing multiple MSC configurations per beam angle. With respect to target coverage, CPO-M provides maximum target dose comparable to IMPT in all cases. These results suggest that optimized MSC positioning through CPO-M can partially mitigate the conformity penalties traditionally associated with SFRT, while preserving its advantages in normal tissue sparing and PVDR.

#### 3.2.2 Comparison with joint dose-PVDR optimization (JDPO) [12]

The proposed methods were compared against JDPO method [12] that simultaneously optimizes dose and PVDR across various slices, while also strategically choosing MSC with different ctc distances at each angle. When compared with the JDPO framework [12], the proposed methods highlight complementary strengths and trade-offs. JDPO consistently achieves the highest PVDR values across all cases, owing to the explicit inclusion of PVDR objectives in its formulation. However, this increased spatial modulation comes at the cost of inferior target dose coverage and higher mean doses to OAR relative to both CPO-S and CPO-M. In contrast, although PVDR is not explicitly optimized in the proposed framework, both CPO-S and CPO-M achieve PVDR values that remain substantially higher than IMPT, while simultaneously improving OAR sparing.

Among SFRT-based methods, CPO-M consistently achieves the lowest mean OAR doses for most OAR across all cases, demonstrating that MSC positioning via lateral shifting provides a more effective mechanism for balancing spatial fractionation and dose conformity than collimator selection strategy [12] alone. Furthermore, CPO-M improves target dose coverage relative to JDPO in all cases, highlighting the benefit of introducing MSC positioning as an additional degree of freedom in inverse pMBRT planning. These results indicate that while JDPO improves spatial modulation (PVDR), the proposed methods, particularly CPO-M, achieve a more favorable overall balance between PVDR, OAR sparing, and target coverage.

#### 3.2.3 Comparison with conventional pMBRT with fixed MSC positioning (Conv)

Relative to the conventional approach with fixed MSC positioning (0 mm shift), both CPO-S and CPO-M consistently improve plan quality across all evaluated cases. Fixed MSC positioning restricts the ability to adapt minibeam locations to patient-specific anatomy, often resulting in suboptimal target coverage and increased OAR doses. In the HN01 case, for example, CPO-S reduced both mean and maximum doses to the oropharynx and larynx compared with the conventional plan, while also lowering the mean dose to the oral cavity. CPO-M provided further reductions in laryngeal doses and improved normalized maximum target dose, reflecting the added benefit of allowing multiple MSC configurations per beam angle. Similar trends were observed across other HN case as well.

In contrast, for abdomen case, improvements in OAR mean doses achieved by CPO-S and CPO-M relative to the Conv method were more modest, suggesting reduced sensitivity to MSC positioning in less anatomically constrained geometries. PVDR values were generally comparable across the three methods, with the conventional and proposed approaches exhibiting higher PVDR values in different beam’s-eye-view slices. Across all anatomical sites, CPO-M consistently provides the most favorable balance among target dose conformity, OAR sparing, and spatial fractionation characteristics. Even in the case with modest absolute gains, CPO-M demonstrates consistent improvements over fixed MSC positioning. Overall, these results highlight MSC position optimization as a meaningful strategy for improving pMBRT plan quality, particularly in anatomically complex clinical cases.

## 4. Discussion

This study highlights the potential of MIP-based optimization (CPO-S and CPO-M) for selecting MSC positions in pMBRT. Allowing MSC positions to be optimized independently for each beam angle enables adaptation to patient-specific anatomical heterogeneity and spatial dose constraints. The results demonstrate that even modest lateral shifts (e.g., ±1 mm) can produce measurable improvements in dose conformity and OAR sparing for certain anatomical sites. Across multiple anatomical sites, MSC position optimization consistently improves plan quality, with the most pronounced benefits observed in anatomically complex cases where the target is closely surrounded by critical structures. In less constrained geometries (abdomen case), the improvements are more modest but remain consistent. Although the magnitude of benefit varies across cases, these findings identify MSC positioning as an important and previously underexplored degree of freedom in pMBRT treatment planning.

The findings in this work complement existing works on beam geometry and collimator parameter optimization in pMBRT. Previous studies have examined the effects of slit width, ctc spacing [12,14], and beam angle selection [34] on treatment quality, demonstrating that adjustments to these parameters can influence beam overlap, spatial modulation, and PVDR in normal tissues. In particular, [14] proposed an MIP-based approach for optimally selecting an appropriate MSC (from a set of pre-manufactured collimators with different ctc distances) for each beam angle, further improving plan quality relative to JDPO [12]. The present work introduces an additional dimension of flexibility by optimizing the lateral positioning of the MSC, enabling spatial adjustment without altering collimator design. This approach is complementary to existing ctc selection strategies [12,14] and suggests a natural extension in which MSC selection (ctc distance) and MSC positioning are optimized simultaneously. Such a unified framework has the potential to further enhance pMBRT plan quality and represents an important direction for future research.

Few limitations of the proposed framework should be acknowledged. First, the mixed-integer optimization introduces additional planning complexity and computational overhead compared with conventional IMPT and fixed-MSC pMBRT planning. However, the reported runtimes remain practical for offline treatment planning. Second, clinical implementation requires accurate and reproducible lateral positioning of the MSC at each beam angle, which may result in a modest increase in setup or delivery time. From a feasibility standpoint, mechanical studies have demonstrated that variable MSC shifts can be performed without mechanical interference or positioning inaccuracies [35,36]. Consequently, the primary trade-off of the proposed approach is a small increase in total treatment time due to MSC repositioning at each angle, which is generally acceptable for high-precision treatments when accompanied by clinically meaningful improvements in OAR sparing. Finally, the magnitude of the dosimetric improvements varies across cases, highlighting the need for validation on larger and more diverse patient cohorts.

Clinical deployment of MSC-based pMBRT with optimized positioning will require dedicated quality assurance (QA) procedures to ensure accurate and reproducible delivery. Key QA considerations include verification of MSC slit geometry and center-to-center spacing, confirmation of lateral MSC positioning accuracy and repeatability, and alignment of the MSC relative to the beam axis for each gantry angle, requirements that are partially addressed by existing mechanical validation studies [35,36]. In addition, high-resolution dosimetric measurements, such as radiochromic film or fine-pitch detector arrays, may be necessary to verify peak-valley dose patterns and spatial fractionation characteristics during commissioning and routine QA. These requirements are consistent with QA practices reported for other spatially fractionated proton delivery techniques and represent an essential step toward clinical translation of the proposed optimization framework within the pMBRT modality.

Finally, it is useful to contextualize the proposed approach within the broader landscape of spatially fractionated radiation therapy. Traditional grid therapy employs centimeter-scale apertures to generate coarse patterns of high- and low-dose regions. pMBRT extends this concept by enabling millimeter-scale spatial fractionation while leveraging the depth-dose characteristics of protons. Compared with grid therapy, MSC-based pMBRT provides improved depth conformity and greater flexibility in shaping peak-valley dose patterns across beam angles. The proposed MSC position optimization framework further enhances this flexibility by enabling beam angle specific modulation of minibeam placement, a degree of freedom not typically available in conventional grid-based approaches. As such, this work complements existing spatially fractionated techniques and highlights the role of inverse optimization in exploiting the unique capabilities of proton-based delivery.

## Supplementary Material

This is a list of supplementary files associated with this preprint. Click to download.

- SMMIPMSCShift020226.docx

## Figures and Tables

**Figure 1:**
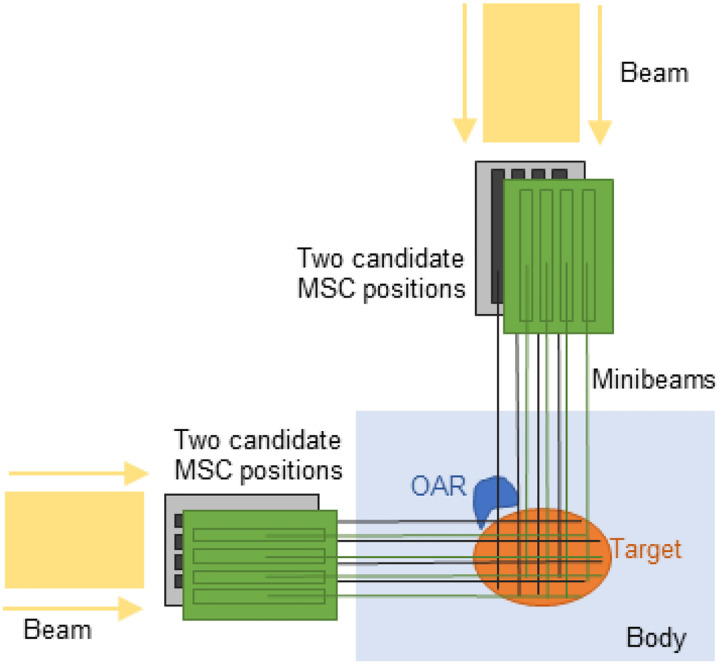
Illustration of the problem formulation. Two beam angles (0° and 270°) are shown. For each angle, two candidate MSC positions are depicted as small lateral shifts relative to the beam direction. For the 0° beam, shifts occur left-right, whereas for the 270° beam, shifts are perpendicular to the plane of the paper. Shifting the MSC alters the peak-valley dose pattern, and appropriate selection of MSC positions can improve target dose uniformity and OAR sparing.

**Figure 3:**
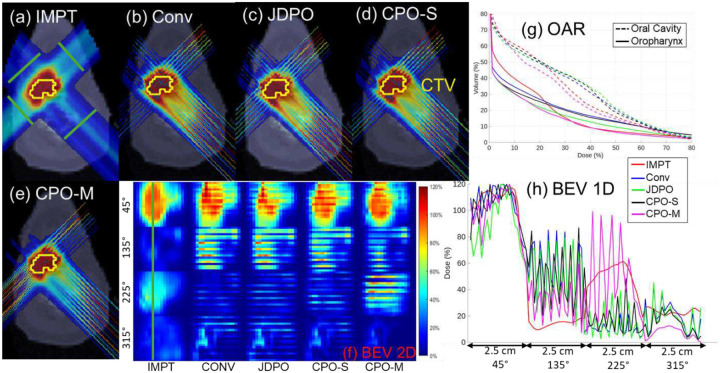
(HN01): (a)-(e) Dose plots for the five methods evaluated. (f) Dose distributions in 2D BEV slices at locations indicated by green lines in (a). (g) OAR DVH plots. (h) Lateral dose profiles at 1D slices marked with green line in (f).

**Figure 4:**
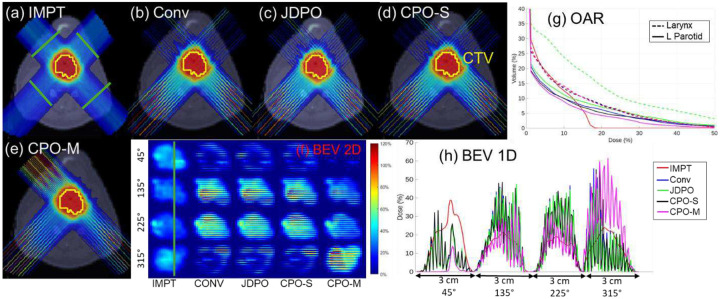
(HN02): (a)-(e) Dose plots for the five methods evaluated. (f) Dose distributions in 2D BEV slices at locations indicated by green lines in (a). (g) OAR DVH plots. (h) Lateral dose profiles at 1D slices marked with green line in (f).

**Figure 5:**
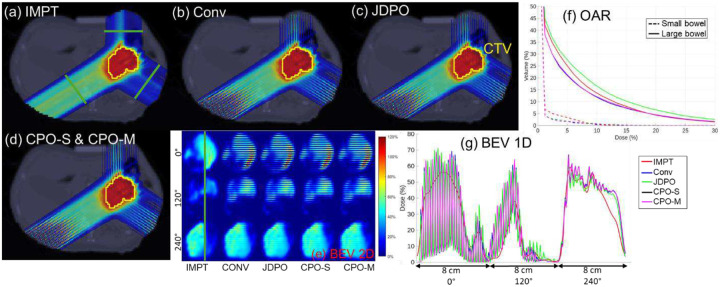
(Abdomen): (a)-(d) Dose plots for the five methods evaluated. (e) Dose distributions in 2D BEV slices at locations indicated by green lines in (a). (f) OAR DVH plots. (g) Lateral dose profiles at 1D slices marked with green line in (e).

**Table 1:** (HN01): Comparison of IMPT, conventional MSC positioning (Conv), JDPO [12], single-MSC MIP optimization (CPO-S), and multiple-MSC MIP optimization (CPO-M). The PVDR and Dmean values are calculated in 2D BEV slices indicated by green lines in Figure 3(a).

		IMPT	Conv	JDPO	CPO-S	CPO-M
Target	Time (secs)	171.43	334.21	410.09	458.11	426.52
	Dmax	128.45%	132.75%	134.86%	132.32%	127.46%
Oral Cavity	Dmax (Gy)	47.74	58.49	57.47	60.63	50.55
	Dmean (Gy)	5.76	6.50	5.24	6.16	4.64
Oropharynx	Dmax (Gy)	40.79	39.72	40.45	39.29	40.32
	Dmean (Gy)	9.90	10.31	10.64	10.45	9.62
Larynx	Dmax (Gy)	42.43	44.36	44.62	44.22	43.09
	Dmean (Gy)	1.13	1.54	1.53	1.40	1.25
Body	Dmean (Gy)	0.26	0.30	0.29	0.28	0.25
	PVDR (45°)	6.17	6.41	5.84	7.23	6.00
	PVDR (135°)	5.04	7.10	6.46	5.89	6.46
	PVDR (225°)	4.91	6.67	9.40	5.60	4.98
	PVDR (315°)	5.07	9.61	12.64	8.25	13.11

**Table 2:** (HN02): Comparison of IMPT, conventional MSC positioning (Conv), JDPO [12], single-MSC MIP optimization (CPO-S), and multiple-MSC MIP optimization (CPO-M). The PVDR and Dmean values are calculated in 2D BEV slices indicated by green lines in Figure 4(a).

		IMPT	Conv	JDPO	CPO-S	CPO-M
Target	Time (secs)	205.19	447.01	441.90	619.98	593.77
	Dmax	112.50%	113.92%	124.72%	112.10%	111.03%
L Parotid	Dmax (Gy)	13.39	57.67	62.42	51.66	41.12
	Dmean (Gy)	1.86	2.19	2.59	2.11	1.74
Larynx	Dmax (Gy)	38.00	40.00	54.48	40.17	40.30
	Dmean (Gy)	2.86	2.80	5.20	2.79	2.78
Body	Dmean (Gy)	0.57	0.61	0.62	0.61	0.57
	PVDR (45°)	6.70	9.03	10.17	9.47	6.02
	PVDR (135°)	5.51	8.62	8.15	9.57	7.61
	PVDR (225°)	4.48	7.89	8.02	7.80	6.01
	PVDR (315°)	7.41	9.79	9.39	9.97	8.44

**Table 3:** (Abdomen): Comparison of IMPT, conventional MSC positioning (Conv), JDPO [12], single-MSC MIP optimization (CPO-S), and multiple-MSC MIP optimization (CPO-M). The PVDR and Dmean values are calculated in 2D BEV slices indicated by green lines in Figure 4(a).

		IMPT	Conv	JDPO	CPO-S	CPO-M
Target	Time (secs)	205.30	505.08	382.63	607.18	591.42
	Dmax	119.77%	123.52%	129.13%	123.31%	123.31%
Large bowel	Dmax (Gy)	23.55	23.81	25.66	24.16	24.16
	Dmean (Gy)	0.97	0.87	1.15	0.87	0.87
Small bowel	Dmax (Gy)	2.98	4.49	5.09	4.04	4.04
	Dmean (Gy)	0.09	0.05	0.06	0.05	0.05
Body	Dmean (Gy)	1.12	1.13	1.09	1.13	1.13
	PVDR (0°)	6.18	13.06	14.95	12.78	12.78
	PVDR (120°)	5.24	11.54	11.28	11.73	11.73
	PVDR (240°)	6.48	8.16	9.13	8.11	8.11

## Data Availability

The datasets generated and analysed during the current study are available from the corresponding author on reasonable request.
